# Factors influencing the reliability of measurements in eyes with full-thickness macular holes: are we measuring incorrectly?

**DOI:** 10.1136/bmjophth-2023-001531

**Published:** 2024-08-16

**Authors:** George Moussa, Assad Jalil, Myrta Lippera, Nouf Alnafisee, Tsveta Ivanova

**Affiliations:** 1 Academic Unit of Ophthalmology, University of Birmingham College of Medical and Dental Sciences, Birmingham, UK; 2 Manchester Royal Eye Hospital, Manchester, UK

**Keywords:** Retina, Macula, Imaging

## Abstract

**Purpose:**

The calliper function is used for manual measurements of full thickness macular holes (FTMHs). We aimed to investigate whether a reproducible difference could be detected beyond interobserver variability between two commonly used manufacturers in their manual calliper facility in spectral domain optical coherence tomography (OCT) for metrics related to FTMH.

**Methods:**

This is a non-interventional, retrospective, observational study. Two independent observers examined 8 eyes (16 OCT) scans and 128 measurements (minimal linear diameter (MLD), basal diameter and hole height on both sides) of FTMHs, taken on Heidelberg Spectralis and Topcon Triton (OCT machines). The interobserver agreement and OCT machine agreement of measurements were analysed by Bland-Altman plots and intraclass correlation coefficient (ICC) analysis. Spectralis and Triton had 125 µm and 50 µm horizontal b-scan spacing, respectively.

**Results:**

Overall, we report high absolute agreement in interobserver (ICC 0.991 (95% CI 0.985 to 0.995, p<0.001)) and OCT machine (ICC 0.993 (95% CI 0.987 to 0.996, p<0.001)) variability. Lower horizontal resolution in Triton compared with Spectralis leads to interobserver variability, in smaller horizontal measurements. Lower horizontal scanning density in Spectralis lead to relatively large interobserver variation if different reference scans were chosen, and consistently smaller MLD measurements than Triton. Vertical measurements without 1:1 scaling lead to inaccurate exaggerated oblique vertical measurements. Calliper function appears otherwise identically calibrated.

**Conclusions:**

We report excellent interobserver and OCT machine agreement in measurements. However, the paper shows several factors that could influence the reliability of measurements acquired in eyes with FTMHs, such as the dimension of the hole as well as different image resolution, density scanning protocols or vertical scaling of the OCT machines viewing platform.

**WHAT IS ALREADY KNOWN ON THIS TOPIC**

- The calliper function is a useful tool for the acquisition of manual linear measurements in eyes with full thickness macular holes (FTMHs). However, no previous study has investigated the reproducible difference beyond interobservers and calibration of callipers in different optical coherence tomography (OCT) manufacturers for metrics related to FTMH.

**WHAT THIS STUDY ADDS**

- Measurements on default settings on modern spectral-domain OCT software lead to significant overestimated readings, especially of vertical measurements relative to true size. The use of 1:1 horizontal:vertical scaling is essential for manual measurements.
- Low-density horizontal b-scans reduce true horizontal measurements, especially in small FTMHs.
- Low resolution leads to difficulties in the identification of the reference points for measuring.

**HOW THIS STUDY MIGHT AFFECT RESEARCH, PRACTICE OR POLICY**

- Although we report excellent interobserver and OCT machine agreement in measurements, caution is advised for the interchangeable use of OCT scans, especially for research purposes, particularly for smaller horizontal measurements.
- We recommend the use of OCT machines with high-resolution, higher-density scanning protocol and 1:1 vertical scaling in order to optimise the reliability of measurements in eyes with FTMHs.

## Introduction

 Optical coherence tomography (OCT), allowing for cross-sectional high-resolution retinal imaging in a non-invasive manner,^1^ is nowadays essential to ophthalmologists for diagnoses, monitoring pathologies and assessing outcomes of treatment.^2^ Moreover, it is also extensively used as a research tool providing objective metrics in clinical trials.^3^ Although it has been previously demonstrated that retinal thickness measurements obtained with various OCT devices are different beyond clinical practice tolerance,^1 2^ the differences were attributed to the analysis algorithms used, differences in software segmentation of retinal layers or reference points between manufacturers.^4^

A full thickness macular hole (FTMH) is defined as an interruption of all the neuroretinal layers in the fovea,^5^ which can achieve a high success rate following surgery with anatomical closure in over 90% of cases.^6 7^ However, different anatomical closure rates have been described based on the minimum linear diameter (MLD) of the retinal defect; the parameter most used in clinical practice in FTMH management due to its role as a predictor for surgical outcomes.^8^ Other less commonly used parameters to predict prognosis following surgery include the basal diameter (BD) and the height of the FTMH.^9^,^11^

To the author’s best knowledge, there are no automated measurements performed by mainstream OCT manufacturers related to FTMH and the OCT-based parameters used in FTMHs are measured by manual selection of the distance of interest. To date, no prior study has investigated whether callipers are calibrated between manufacturers in manual measuring of metrics on an OCT scan in FTMH measurements. As manual measurements are integral to clinical work related to FTMH, and research related to the topic (particularly for studies and centres that use more than one type of OCT machine), we aimed to investigate whether a reproducible difference could be detected beyond interobserver variability between two commonly used manufacturers in their manual calliper facility in spectral domain (SD) OCT for metrics related to FTMH.

## Methods

We present a single-centre, retrospectively, non-interventional observational study to investigate reproducibility, beyond interobserver variability, of measurements between two commonly used OCT machines in eyes awaiting surgery for FTMH at the Manchester Royal Eye Hospital. Patients were identified from waiting lists for upcoming FTMH surgery and anonymised on extraction.

The patients included in the study underwent imaging using two different SD-OCT machine on the day of surgery, preoperatively: Heidelberg SPECTRALIS HRA+OCT (Spectralis SD-OCT; Heidelberg Engineering, Heidelberg, Germany) and Topcon DRI OCT Triton (Topcon, Tokyo, Japan). Following pharmacological mydriasis, all patients underwent the acquisition protocol of the machine for macular volume, with the distance between consequential horizontal b-scans of 125 µm and 50 µm, respectively, for Spectralis and Triton.

After two graders completed an initial training session of measurement technique, they independently acquired the measurements. For each eye, four parameters were measured, using the calliper tool integrated into the machine, for the linear OCT scan crossing as close as possible to the centre of the fovea: MLD is defined as the horizontal minimum hole diameter in the OCT slice with the widest dimension; BD is defined as the horizontal diameter at the level of the retinal pigment epithelium in the OCT slice with the widest dimension and hole heigh is defined as the greatest height from the retinal pigment epithelium to the vitreoretinal interface at the nasal and temporal side of the FTMH.^12^ All measurements were calculated using the default viewing platform for each machine.

### Statistical analysis

All statistical analyses were performed by using IBM SPSS Statistics for Windows, V.29.0 (IBM). Statistical significance was defined as p<0.05. Interobserver variation and difference between OCT machine measurements were assessed with Bland-Altman plots^13^ along with computation of the intraclass correlation coefficient (ICC) and interobserver correlation coefficient using a two-way mixed-effects model for measurements of absolute agreement. For comparing the variability of OCT machines, the mean measurement of the two observers was used.

## Results

In our cohort, we report on the comparison of 128 (64 for each observer) manual measurements performed across 8 eyes of 8 patients (16 OCT scans, 8 each from Spectralis and Triton, respectively).

### Interobserver variability

**Figure 1:**
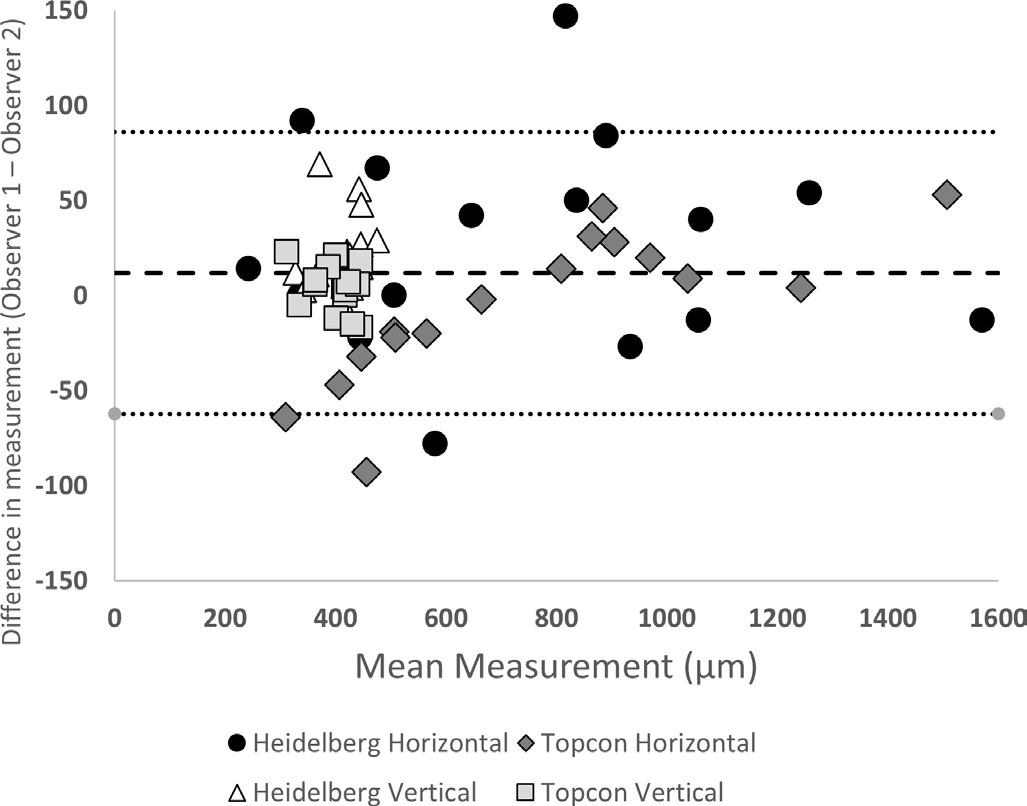
Bland-Altman plots of agreement for interobserver variability across all measurements. Bland-Altman plots of interobserver agreement. There appears to be wide variability particularly in smaller measurements for horizontal Heidelberg Spectralis measurements. Observer 1 systematically measured smaller values than observer 2 for Triton horizontal measurements, particularly in lower values. The interclass correlation coefficient 0.991 (95% CI 0.985 to 0.995, p<0.001).

**Figure 2:**
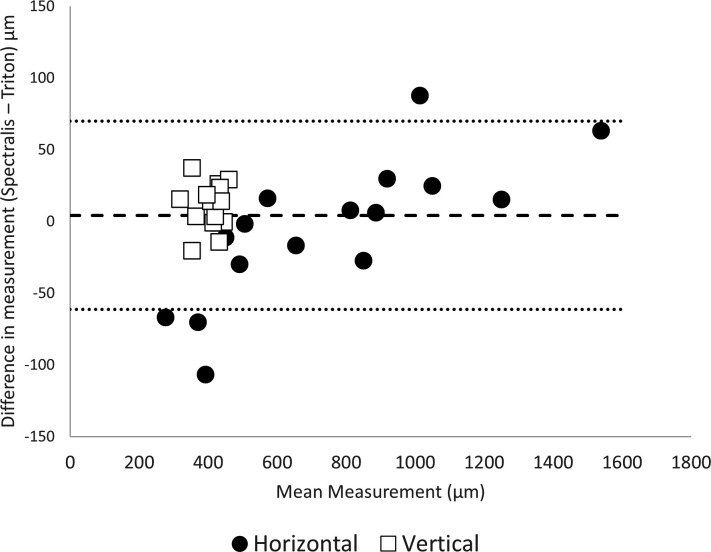
Bland-Altman plots of agreement for OCT machine variability across all measurements. Bland-Altman plots of OCT machine measurement variability. Spectralis relative to Triton consistently had smaller horizontal measurements, particularly for smaller measurements. Heidelberg had consistently higher measurements relative to Triton for vertical measurements. The interclass correlation coefficient 0.993 (9% CI 0.987 to 0.996, p<0.001). OCT, optical coherence tomography.

**Figure 3:**
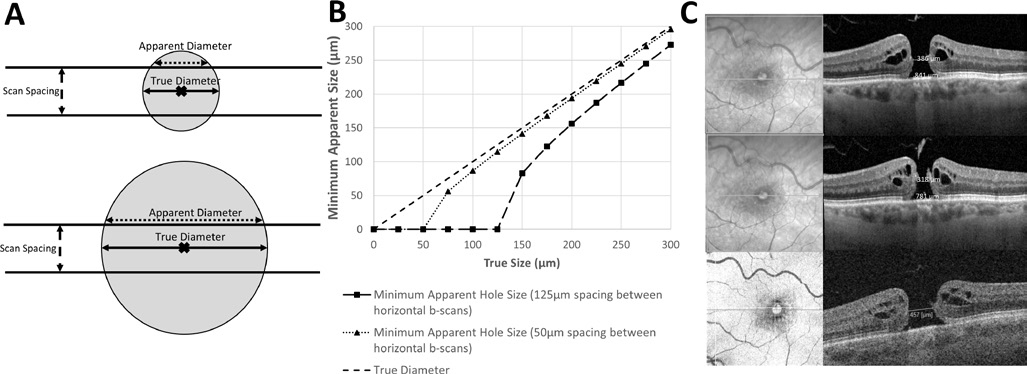
Effect of scanning density on the minimum possible apparent horizontal measurement size. (**A**) For the same horizontal b-scan spacing (scan density), smaller macular holes may appear relatively smaller than larger macular holes. (**B**) The relationship between minimum possible apparent size, scan density and true diameter (eg, MLD) is $MinimumApparentSize=\sqrt{TrueDiameter^{2}-{ScanSpacing}^{2}}$. The Spectralis scan density was 125 µm between horizontal b-scans while Triton had 50 µm spacing. (**C**) The top two figures represent Spectralis through the same macular hole with clinicians choosing different horizontal slices measuring an MLD of 386 µm and 318 µm. The bottomscan represents the same macular hole with Triton with a horizontal slice better through the true midpoint of the macular hole, being more representative of the true MLD (457 µm). MLD, minimal linear diameter.

**Figure 4:**
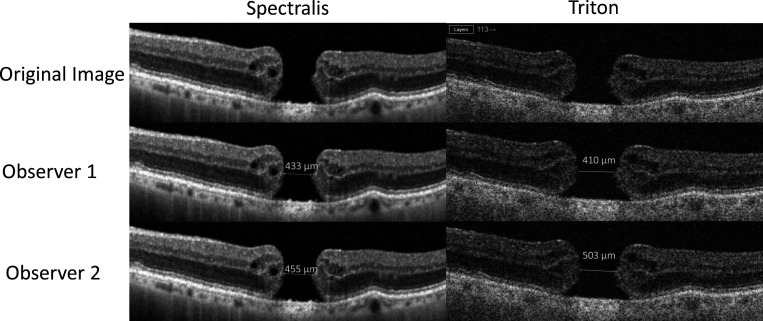
Effect of resolution on reference points. The superior lateral and axial resolution of Spectralis over the Triton is evident. Observers 1 and 2 chose different reference points to measure the minimum linear diameter particularly on the right border. This right border is less defined in the Triton scan, leading to a difference of 93 µm with the Triton and 22 µm with Spectralis.

Figure 1 represents Bland-Altman plots of agreement for interobserver variability across all measurements, the data points differentiate between the different OCT machines used and horizontal or vertical measurements. For the whole cohort, there is a high level of agreement between observers (ICC 0.991 (95% CI 0.985 to 0.995, p<0.001)). However, scrutiny of the Bland-Altman plots differentiating the OCT machines and observers identified two main areas of measurement bias: on one hand, wider variability could be described in smaller horizontal measurements obtained with Spectralis (figure 2); on the other hand, observer 1 consistently measured lower distances than observer 2, for horizontal measurements obtained with Triton, in case of acquisition of smaller values (ie, MLD rather than BD) (figure 1). Independent from the OCT machine used, variability was detected in the case of smaller FTMH (figure 3): indeed, if the horizontal b-scan does not transect the FTMH directly through the centre, which might happen more frequently in case of low scanning density, the horizontal MLD and BD measured from the other scans will be smaller than their real measurements. As observer 1 consistently measured smaller distances than observer 2 in the scans acquired, we revisited all the imaging and measurements taken with Triton. We found that in many cases, observer 1 and observer 2 had selected different reference points in the OCT scan (figure 4) for scans acquired with Triton, mainly due to ambiguity due to poor resolution of the imaging.

### Impact of the scan density on the measurements

Interobserver variability is particularly evident in the case of a lower scanning density. The larger the spacing between horizontal b-scans (lower scanning density) by the OCT machines, the larger the possible discrepancy between measured size and true size. With the scanning protocol used, Spectralis takes fewer scans (49 horizontal b-scans separated by 125 µm), compared with Triton (149 scans separated by 50 µm), therefore, the reference image observers choose to perform measurements, with fewer horizontal b-scans will result in larger interobserver variation in horizontal measurements as evidenced in figure 1 (wide interobserver variability in Spectralis horizontal measurements compared with Triton horizontal measurements). We demonstrate and calculate the effect of horizontal b-scan scanning density on the acquisition of MLD measurement, and therefore, on the minimum possible apparent horizontal measurement size (figure 3). In figure 3B, we plotted the minimum possible measurement relative to true measurement size depending on b-scan spacing (scan density); smaller FTMHs are most affected by this phenomenon (figure 3C).

### OCT machine variability

Figure 2 is a Bland-Altman plot of OCT machine measurement variability. Spectralis, relative to Triton, consistently had smaller horizontal measurements, especially for smaller measurements. Spectralis had consistently higher measurements relative to Triton for vertical measurements. However, overall, there was a high absolute agreement between both OCT machines (ICC 0.993 (95% CI 0.987 to 0.996, p<0.001)).

**Figure 5:**
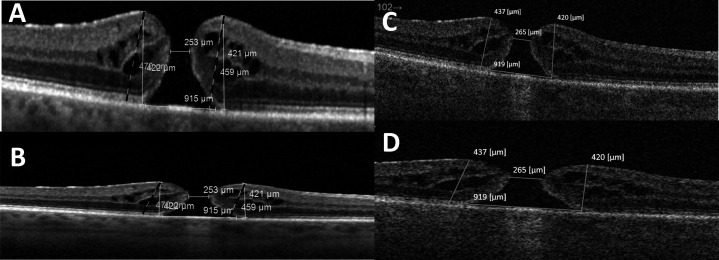
Effect of vertical scaling on measurements The figure shows the linear OCT scan, crossing the centre of the fovea, performed on the same eye using Spectralis (**A–B**) and Triton (**C–D**). In figure 4A, the default vertical scaling (1:1 pixel, equivalent to horizontal:vertical scaling of 1:3.775 µm) of Spectralis is set, which was modified in figure 4B to 1:1 µm. Similarly, in figure 4C, the default vertical scaling (1:2 µm) of Triton is applied, while in figure 4D, the scaling was modified to 1:1 µm. The dashed lines in figure 4A,C represent hole height measurements taken by the observer using the default viewing scale for Spectralis and Topcon. In figure 4A, left and right hole height (HH) are measured as 470 µm and 459 µm, respectively, using Spectralis, while in figure 4C, left and right HH are measured 437 µm and 420 µm, respectively, using Topcon, resulting in a difference measuring the same parameters between the two OCT machines. However, figure 4B demonstrates that following changing the scaling to 1:1 µm, the original dashed vertical measurements are oblique and therefore invalid: white lines have been redrawn, on the 1:1 µm viewing scaling, which represent the new and correct vertical measurements (left HH 422 µm and right HH 421 µm). Differently, the Topcon IMAGENet viewing system by default employs a 1:2 horizontal:vertical scaling (less than Spectralis). Therefore, although oblique dashed lines are still evident after changing the scaling to 1:1 µm, they are less apparent. Horizontal measurements are minimally affected by this phenomenon and appear consistent regardless of vertical scaling, unless measured obliquely.

Spectralis and Triton have different default vertical scaling in their viewing system. Triton by default in their IMAGENet viewing system have a 1:2 ratio (horizontal:vertical) so that vertical distances appear twice as large as horizontal distances. The Spectralis default viewing system has a higher scaling at 1:3.775.^14^ These have implications for vertical measurement as described in figure 5. We demonstrate vertical measurements performed with scaling and then the same images with no scaling (1:1). With an FTMH height measurement performed at a horizontal:vertical scaling greater than 1:1, it is possible to accidentally measure obliquely rather than the true FTMH height perpendicular to the retinal pigment epithelium, resulting in larger than true measurements.

Following data collection and analysis, when OCT images were purposefully compared, side by side from Spectralis and Triton images of the same eye, the callipers appeared to measure identically (figure 5).

## Discussion

In this study, we aimed to analyse the compatibility of two SD OCT machines for manual measurements of FTMH, determining whether they could be used interchangeably for clinical and research purposes. Although we found that, on careful side-by-side comparisons, the calliper function appears to be calibrated identically between both OCT software; there are several factors that lead to differences depending on the OCT scanning protocols and measurement techniques which are configurable on both machines.

Optical resolution in OCT has been defined as the ability of the machine to distinguish two closely spaced points as physically different from a single point.^15^ In the scientific literature, a lower resolution has been proposed as an important cause of variability when comparing OCT measurements with different machine^15^,^17^ while other studies stated that the differences in measurements could not be fully explained by a lower resolution.^1 18^ In our study, we confirm that the differences in resolution resulted in different reference points for measuring. Indeed, observer 1 repeatedly measured less than observer 2 for small horizontal distances such as MLD relative to measurements of OCTs performed with Triton (figure 1). The same did not happen with a machine with higher resolution, as Spectralis offers 5.7 µm/pixel lateral resolution and 3.9 µm/pixel axial resolution,^19^ and Triton has 20 µm lateral resolution with in-depth digital and optical function resolution of 2.6 µm and 8 µm, respectively.^20^ As shown in figure 4, the two observers measured differently the MLD, due to lower lateral resolution on Triton. Differently, there was less ambiguity in reference points while measuring BD: no difference was found in the larger horizontal measurements between observers on the Bland-Altman plot (figure 1).

Another contributor to interobserver variability was the selection of different OCT horizontal ‘slices’; particularly evident in smaller FTMH and in larger spacing between scans. In the case of the same protocol of OCT acquisition, and therefore, the same scanning density, interobserver variability is higher for smaller FTMHs. Indeed, as shown in figure 3A, the absence of an OCT linear scan that dissects the FTMH exactly at its maximum diameter, frequently in patients with poor vision or eccentric fixation; a wider difference could be observed in smaller FTMHs between apparent (or measured) and true (or anatomical) MLD or between MLDs measured by two observers. This could be explained as they chose to acquire measurements from distinct linear scans. In the case of OCT acquisition of the same, or equally sized, FTMH; higher interobserver variability could be observed for protocols with larger spacing between horizontal b-scans. Indeed, higher-density scans allow better possibility to dissect the FTMH in its maximum diameter, or to detect a smaller difference between apparent and true MLD or between MLDs measured by two observers who chose to acquire measurements from distinct linear scans. This may happen despite the observers’ efforts to choose the widest diameter to measure, which is not always obvious even for experienced observers, particularly in high myopes and oblique scans. This is evident in the wide interobserver variability in horizontal measurements for Spectralis relative to Triton (figure 1), due to its lower scan density (49 horizontal b-scans separated by 125 µm vs 149 scans separated by 50 µm of Triton). For the same reason, figure 3B shows why Spectralis resulted in consistently lower horizontal measurements than Triton (figure 2) particularly for small FTMH, as a small MLD is most sensitive to undermeasurement relative to true width (figure 3B). Different raster scans are available for OCT machines, including horizontal and vertical grid patterns; high-density raster scans, with higher spatial sampling and finer details compared with standard patterns. This is offset by longer acquisition time, increased patient compliance, effective eye tracking facility and higher storage. In addition, the information technology infrastructure may not enable loading high-density scans in day-to-day clinical practice. As such, a balance must be struck between various image acquisition parameters and practicality. Macular radial scans are rapid and consist of a series of radial scan lines originating from the fovea and extending outward in different directions. Among the different types of scans, radial OCT scans have been previously described in the scientific literature as a better option for assessing FTMH.^21^ Indeed the use of a radial raster could potentially better find the true maximum diameter in case of irregularly shaped FTMHs. However, in case of poor vision/fixation and low density of scans, if the geometric centre of the radial raster is not exactly allocated in the centre of the FTMH, measurement accuracy and precision could be reduced. Differently, the present study demonstrates the importance of high-density raster scans in order to reduce the difference between apparent and true diameter, as well as the interobserver variability.

Finally, a high vertical-to-horizontal scaling is useful in clinical assessment as it allows for vertical magnification of retinal layers. However, as demonstrated in figure 5, this may result in highly erroneous vertical measurements, leading to accidental oblique measurements that are obvious only when viewing images in 1:1 scaling, and therefore, exaggerating vertical height measurements. As Spectralis has higher scaling than Triton by default, this resulted in higher vertical measurements relative to Triton (figure 2). When both software viewing systems were set to 1:1 scaling and measurements repeated, this led to identical vertical measurements between the different OCT machines. Garcia Garrido *et al* discussed the issue of OCT scaling in murine eyes and advised equal scaling to improve the efficiency of OCT image analysis.^22^ To the author’s best knowledge, the issue of vertical measurement error due to scaling has not been previously discussed in the literature pertaining to FTMH measurement and we speculate that most researchers measure on the default viewing setting, due to the lack of discussion in the methodology of such papers. Although our paper primarily discusses measurements for FTMH, the scaling vertical measurement effect will be applicable to any condition that requires manual vertical measurements on OCT. An example of this is the thickness of acute submacular haemorrhage secondary to underlying choroidal neovascular membrane, where the vertical height of the haemorrhage at the fovea can be a determinant of the management strategy. There is evidence that smaller bleeds especially with a thickness less than 450 µm do well with anti-VEGF monotherapy, as against a combination treatment of anti-VEGFs with expansile gas and tissue plasminogen activator used in thicker bleeds.^23^ As such, we strongly recommend that all vertical measurements are performed with 1:1 scaling (labelled 1:1 µm as opposed to 1:1 pixel in Spectralis), especially in cases with clinical implications.

In the scientific literature, other studies have compared intravariability and intervariability in measurements of MLD or BD in FTMH between different observers using the same machine (Spectralis or Topcon), finding no significant difference.^24 25^ Similarly, Chen *et al* reported no significant difference for MLD (p=0.69) in FTMH detected between two observers using Spectralis^11^; however, a significant difference in measurements of BD (mean of the differences 38.75 µm, with observer 2 overestimating BD compared with observer 1; p<0.0001) was described.^11^ This is the first study to compare the compatibility of two different OCT machines in the acquisition of measurements for FTMHs. Although we describe sources of error and their effect on interobserver variability, we find a very high ICC between both observers and OCT machines.

The limitations of our study include its reduced sample size of 128 measurements in 8 eyes across 2 observers. However, these data were sufficient to highlight sources of repeatable variability between OCT machines. Finally, the scan protocols used were standard protocols used in our clinical settings and higher-density scan protocols, although available, were not acquired. The study evaluated exclusively horizontal grid patterns and did not take in consideration other OCT raster patterns such as the radial one. Horizontal patterns can measure the maximum diameter of an FTMH, which might not be the true MLD in case of irregular shaped FTMH. Finally, the two OCT protocols compared use different horizontal:vertical ratio, both of which can be set to identical scaling. Scans with higher density with Spectralis and 1:1 horizontal:vertical ratio would mitigate many of the differences in measurements we address in the paper.

In conclusion, we find good interobserver and OCT machine agreement in measurements with identically calibrated calliper functionality. However, the paper highlights possible factors that could influence correct measuring and interobserver variability for acquisition of quantitative linear parameters in FTMH: resolution, density scanning protocol of the OCT machine and vertical scaling when measuring, as well as dimension of the hole. Therefore, to increase the reliability of measurements in eyes with full-thickness macular holes, we recommend optimal, use of a a higher scanning density protocol, possible with most OCT manufacturers, to minimise the error caused by the selection of the incorrect/different OCT linear scar dissecting the centre of the FTMH, with an OCT device that enables high resolution, to better identify the reference points for measuring. In the case of acquisition of vertical measurements, 1:1 horizontal:vertical scaling should be deployed as a or postacquisition modification before taking the measurements, to reduce exaggerated vertical measurements relative to true size. Finally, awareness that caution is advised for the interchangeable use of OCT scans for research purposes, particularly for smaller horizontal measurements.

## supplementary material

10.1136/bmjophth-2023-001531 online supplemental file 1

## Data Availability

Data are available on reasonable request.
